# Utilizing augmented artificial intelligence for aminoacidopathies using collaborative laboratory integrated reporting- A cross-sectional study

**DOI:** 10.1016/j.amsu.2022.104651

**Published:** 2022-09-23

**Authors:** Zaib Un Nisa Khan, Lena Jafri, Patricia L. Hall, Matthew J. Schultz, Sibtain Ahmed, Aysha Habib Khan, Hafsa Majid

**Affiliations:** a Resident Chemical Pathology, Department of Pathology and Laboratory Medicine, Aga Khan University, Pakistan; b Department of Pathology and Laboratory Medicine, Aga Khan University, Pakistan; c Mayo Clinic Department of Laboratory Medicine, and Pathology, Rochester, Minnesota, USA

**Keywords:** Amino acids, inherited Metabolic disorders, Maple syrup urine disease, Phenylketonuria, Artificial intelligence

## Abstract

**Introduction:**

Plasma amino acids profiling can aid in the screening and diagnosis of aminoacidopathies. The goal of the current study was to analyze and report the metabolic profiles of plasma amino acid (PAA) and additionally to compare PAA-reference intervals (RI) from Pakistan with more countries utilizing Clinical Laboratory Integrated Reports (CLIR).

**Methods:**

This was a cross sectional prospective single center study. Twenty-two amino acids were analyzed in each sample received for one year at the clinical laboratory**.** Data was divided into reference and case data files after interpretation by a team of pathologists and technologists. All PAA samples were analyzed using ion-exchange high-performance chromatography. The CLIR application of Amino Acid in Plasma (AAQP) was used for statistical analysis for both data sets and post-analytical interpretive tools using a single condition tool was applied.

**Result:**

The majority of 92% (n = 1913) of PAA profiles out of the total 2081 tests run were non-diagnostic; the PAA values were within the age-specific RI. The PAA median was in close comparison close to the 50th percentile of reference data available in CLIR software. Out of the total 2081 tests run, one hundred and sixty-eight had abnormal PAA levels; 27.38% were labeled as non-fasting samples, and the main aminoacidopathies identified were Phenylketonuria and Maple Syrup Urine Disorder.

**Conclusion:**

An agreement of >95% was observed between the reporting done by the pathologists and technologists’ team and then after the application of CLIR. Augmented artificial intelligence using CLIR can improve the accuracy of reporting rare aminoacidopathies in a developing country like ours.

## Highlights

• Plasma amino acids helps in diagnosing and monitoring of various aminoacidopathies.
• Few aminoacidopathies present with a grossly abnormal investigation profile, with few diseases having subtle deviations.
• Their is 98% concordance of diagnosis concordance of diagnosis of aminoacidopathies between our lab and CLIR.
• CLIR tools can be utilized in a newborn screening program for screening and diagnosis in future.

## 1 Introduction

The analysis of amino acids in body fluids is important for the diagnosis and monitoring of aminoacidopathies as well as in investigating organic acidemias and urea cycle defects. Over 50 different disorders can be diagnosed with the help of a plasma amino acid (PAA) profile, some disorders present with a grossly abnormal profile, while few diseases have subtle deviations [[1], [2], [3]]. Clinical interpretation of a PAA is therefore based not only on the concentrations of individual amino acids but on ratios of different amino acids and an assessment of the overall pattern of the complete PAA profile [[3], [4], [5], [6]]. An approach to refine the interpretation of PAA is using Clinical Laboratory Integrated Reports (CLIR, formerly Region 4 Stork) a post-analytical software that compares the amino acid results against a large database of reference ranges, disease-specific cutoffs, and ratios of diagnostic markers [7,8]. This can be achieved through CLIR using artificial intelligence. In contrast to the cut-off-based approach, this CLIR analysis improves the accuracy and reduces the false results [9,10].

The primary objective of the current study was to interpret complex metabolic profiles of PAA using CLIR. Clinical laboratories in our country refer to reference values of PAA either from kit inserts provided by the manufacturers or from the scientific literature, which are derived from Caucasians. Therefore, the secondary objective of this study was to compare reference intervals (RI) of PAA data from our country to data from other countries with the help of CLIR.

## 2 Materials and method

This was a prospective cross-sectional study, conducted in the Biochemical Genetics Laboratory (BGL) of Section of Chemical Pathology, Department of Pathology and Laboratory Medicine, Aga Khan University (AKU), Karachi Pakistan. The study was carried out after seeking approval from AKU's ethical review committee (Reference number 2019-1709-4536). In accordance with the Declaration of Helsinki, the study was registered with ClinicalTrials.gov research registration database with registry #NCT05437445 https://register.clinicaltrials.gov/prs/app/action/SelectProtocol?sid=S000C93M&selectaction=Edit&uid=U0005Z5D&ts=54&cx=-y6cu9r. The work has been reported in line with the STROCSS criteria [11].

### 2.1 Data collection

Data of all those who had PAA analyzed at the BGL of symptomatic as well as asymptomatic children with suspicion of Inherited Metabolic Disorders (IMDs), from August 2019–August 2020 were included. Plasma amino acids analysis was performed using cation-exchange high-performance liquid chromatography (HPLC) on Biochrom 30+ model using lithium column diameter 4.6 mm and detected at 440/570 nm. The biochemical analysis follows stringent internal quality assurance in line with the Clinical and Laboratory Standards Institution (CLSI) guidelines and the external assessment is ensured and accredited by European Research Network for Evaluation and Improvement of Screening, Diagnosis, and Treatment of Inherited Disorders of Metabolism (ERNDIM) and College of American Pathologists (CAP), respectively. Each identified amino acid profile included two covariates: age at the time of sample collection in years, and gender. Subjects with an age greater than 16 years were excluded from the study. Additionally, the following exclusion criteria were applied to minimize skewing the count of cases with abnormal results based on cutoff-based interpretation [1]: missing covariates [2]; marker results shown as zero, and [3] negative values.

### 2.2 CLIR and its analytical features

CLIR (https://clir.mayo.edu) is an interactive web-based tool developed by the Mayo Clinic in Rochester, Minnesota USA. CLIR maintains a database of laboratory data shared by international sites for aiding post-analytical analysis and is free to utilize with the contribution of data. In 2004, it was developed as multivariate pattern recognition second-generation software to support Region 4 Stork (R4S), which was a performance improvement project aimed to improve newborn screening performance using tandem mass spectrometry. CLIR has an extensive database of confirmed cases and reference data which can be utilized to identify abnormal PAA profiles by comparison to confirmed cases rather than by simple deviation from the reference range [10,12]. Its technique is based on the principles of worldwide laboratory collaboration, data sharing, comparison with peers, and post-analytical interpretive update tools that will be personalized according to the needs of clinicians and laboratory technicians [13,14]. This software allows patient values to be adjusted based on covariates such as age at the time of sample collection and compares them to continuously moving percentiles, instead of traditional discrete benchmarks [12,15]. The moving percentiles are estimated from a large set of normal data provided by participating sites in the CLIR database, illustrating the dynamic pattern of physiological variation for any marker over a wide-ranging covariate [8,12,15,16].

PAA profile along with relevant biochemical and clinical data was reviewed by more than two relevant BGL experts. These results were then compiled in a comma-separated values (.csv) file inclusive of Logical Observation Identifiers Names and Codes (LOINC®) and covariates (age at the time of sample collection in years, gender). Each case was assigned a unique code separate from any other traceable identifier. Two kinds of data were submitted to CLIR: reference data i.e., data of patients with no known metabolic conditions, and cases i.e., data of positive patients or patients with suspicion of IMD. Both files were uploaded to CLIR for analysis by using CLIR data upload functionality. The CLIR application used for statistical analysis was AAQP (Amino Acid in Plasma) for both reference data and cases. CLIR has different types of post-analytical interpretive tools across a wide range of laboratory tests. Using a single condition tool our cases were compared to cases available from all over the world. The single condition tool integrates multiple analytes into a single score, which is assessed against a threshold of clinical significance and when found informative represents the degree of possibility of disease. These tools are created using profiles of confirmed cases and allow for results to be compared against known disease profiles rather than just deviations from a defined reference range. Specific informative markers and calculated ratios are integrated into all tools which provide a score below or above a threshold of clinical significance and a likelihood of disease expressed as a percentile rank in comparison to known cases, described as possibly, likely, and very likely to be seen with the targeted condition.

Frequencies of qualitative variables and mean (SD), and median (Q3-Q1) for quantitative variables were calculated. Data were stratified according to age as follows: neonates (less than 1 month of age), infants (1 month-1 year), toddlers (1–4 years), childhood (4–11 years), and adolescents (>11 years). Concordance was analyzed for cutoff-based interpretation of amino acids versus CLIR interpretations.

## 3 Results

A total of 2083 samples of PAA were analyzed in one year. Out of the total two subjects were excluded as being greater than 16 years of age. The median (Q3-Q1) age of study subjects (2081) was 1 (2-0.16) years. Amongst the total subjects, 56.5% were males (n = 1176). Majority of the subjects were infants (n = 880; 42.2%), followed by neonates (n = 475; 22.8%), toddlers (n = 548; 26.3%), childhood (n = 174; 8.4%) and adolescents (n = 4; 0.2%).

### 3.1 Reference data

**Fig. 1:**
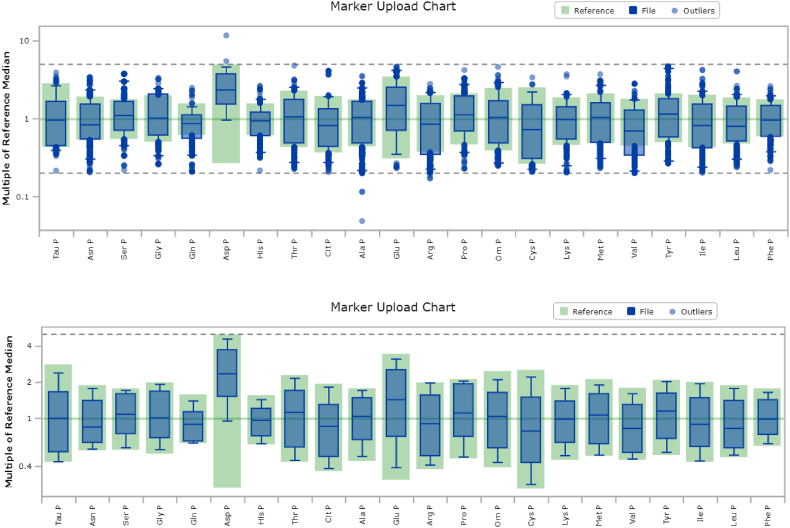
Comparison of plasma amino acid levels reported as ‘Normal Profile’ with reference data using CLIR Data Validation Tool.; Pre-outlier Removal (Fig. 1A) and post-outlier removal (Fig. 1B) ***Legend****: On the x-axis are the twenty-two amino acid and on the y-axis is the Multiple of reference median, where Taurine, Tau; Asparagine, Asn; Serine, Ser; Glycine, Gly; Glutamine, Gln; Aspartate, Asp; Histidine, His; Threonine, Thr; Citrulline, Cit; Alanine, Ala; Glutamate, Glu; Arginine, Arg; Proline, Pro; Ornithine, Orn; Cystine, Cys; Lysine, Lys; Methionine, Met; Valine, Val; Tyrosine, Tyr; Isoleucine, Ile; Leucine, Leu; Phenylalanine, Phe*Removal of the outliers is executed by selecting an interactive function of CLIR called Outlier Removal. All data above and below the 99th percentile and 1st percentile of the reference range, respectively, were shown individually as outliers (blue dots) and were removed. The high and low thresholds to consider a marker value to be an outlier are shown as a grey dotted lines above and below the central part of the plot. The line above is equal to 5 multiples of the cumulative median, and the line below is equal to 0.2 (one-fifth) multiples of the cumulative median, respectively. The green color box and whisker plots represent Amino acid reference data from all over the world and the blue box and whisker plots represent the cumulative data distribution of the present study's population for each amino acid. The middle line over the y-axis represents the 50th percentile or median value for the reference data from all over the world. . (For interpretation of the references to color in this figure legend, the reader is referred to the Web version of this article.)

Out of the total 2081 subjects, 92% (n = 1913) PAA profiles were completely normal with all 22 amino acid values falling within the age-specific reference range. This data (n = 1913) was submitted to CLIR as reference data comprising 1077 males (56.2%). Fig. 1 shows the marker upload chart for all the amino acids studied which displays box plots of each marker range contained within the uploaded file relative to the cumulative reference range, plotted as a multiple of the cumulative reference range median before (Fig. 1a) and after removal of outliers (Fig. 1b). No profile had all values classified as outliers, so the total count of samples remained the same but counts by individual markers inevitably differ after the removal of outliers as shown in Table 1. The majority of PAA median with interquartile range was close to the 50th percentile of reference data except aspartate having 50th percentile 3 times greater than the reference as shown in Table 1.

**Table 1:** Comparison of the median of plasma amino acid reported ‘normal’ at Biochemical Genetics Laboratory of Aga Khan University in comparison to reference data in CLIR after outlier removal.

Marker, n	Median (nmol/L)	
	Present Study	CLIR Reference
Alanine, 1559	344	329.15
Arginine, 1642	58	64
Asparagine, 1633	41.7	49
Aspartate, 1738	8.99	3.84
Citrulline, 1812	20.7	24.2
Cystine, 1748	18.2	23.1
Glutamine, 1628	525	588.3
Glutamate, 1810	69.5	48.5
Glycine, 1566	244	242.5
Histidine, 1672	70.9	74
Isoleucine, 1638	49.5	55.6
Leucine, 1706	82	99
Lysine, 1766	140	142
Methionine, 1686	23.52	22
Ornithine, 1742	58.8	56.3
Phenylalanine, 1659	49.4	50
Proline, 1799	186	168
Serine, 1712	128	118.3
Taurine, 1811	60.2	60.4
Threonine, 1742	122	108
Tyrosine, 1665	65.7	56.9
Valine, 1453	161	195

N = number of subjects after removal of outlier.

**Fig. 2:**
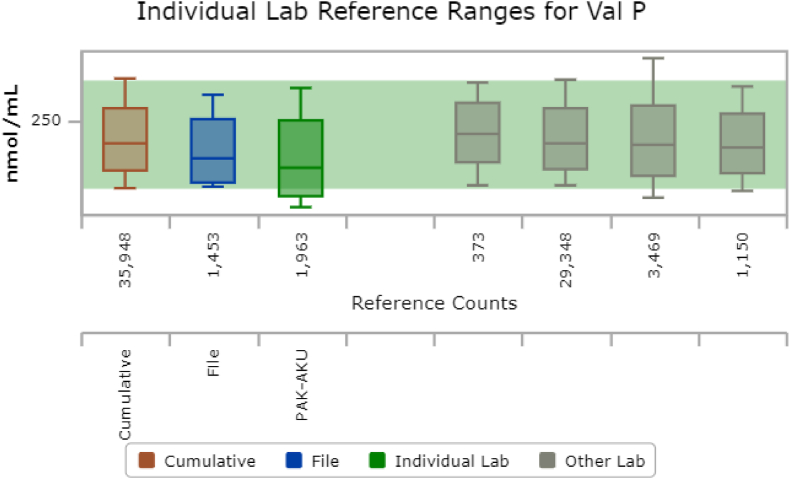
Individual reference range in nmol/ml for plasma valine reported at Biochemical Genetics Laboratory from Aug 2019 to Aug 2020 in comparison to cumulative reference interval data in CLIR Legend: X-axis represents the number of reference count from our country in green color code (individual lab) and blue color code (file) represents the current research file and other labs in grey color code along with cumulative range represented by brown color code and y-axis represent the reference range for plasma valine in nmol/ml. Val P, plasma valine. (For interpretation of the references to color in this figure legend, the reader is referred to the Web version of this article.)

The majority of medians of PAA were in close comparison to the cumulative reference range median except for a few amino acids i.e., cystine, glutamine, and valine (Fig. 2) in which the median was low in comparison to the cumulative reference range amino acids.

### 3.2 Cases data

One hundred and sixty-eight subjects had abnormal PAA profiles with either one or more amino acid concentrations beyond (high or low) the locally defined age-specific reference ranges, shown in Table 2. On application of single condition tools in CLIR 27.38% were identified as non-fasting specimens (NON-FAST), 22% as phenylketonuria (PKU), 10.1% as maple syrup urine disease (MSUD), 8.9% as non-ketotic hyperglycinemia (NKHG), 7.7% homocystinuria as (HCY), 7.14% as elevated glycine (GLY), 4.76% as citrullinemia (CIT-I), 1.8% as carbamoyl phosphate synthetase I deficiency (CPS), and 1.85% as low branched-chain amino acid (BCAA LOW) respectively. Furthermore, the frequency of elevated Proline (PRO), lysinuric protein intolerance (LPI), low methionine (MET LOW), propionic acidemia (PA), and undetermined liver disease (LIVER) were two of each (1.2%). The frequency of the following disorders was one each (0.6%): Heterozygote for ornithine transcarbamylase deficiency (OTC-het), methylenetetrahydrofolate reductase (MTHFR), possible hyperammonemia (GLN) & tyrosinemia-I (TYR-I). Table 2 describes the informative high amino acid markers and interpretation of the CLIR single condition tool.

**Table 2:** CLIR Tool interpretation of informative amino acids markers with the frequency of metabolic disorders identified with CLIR single condition tool from August 2019 to August 2020 in subjects tested at Biochemical Genetics Laboratory of Aga Khan University (n = 168).

Current diagnosis	Informative High Amino acid marker	Total count (%)	CLIR TOOL Interpretation guideline		
			Possibly	Likely	Very likely
NON-FAST	Valine, Isoleucine, Leucine, Phenylalanine, Alanine, Lysine, Methionine, Tyrosine	46 (27.38)	26	19	01
PKU	Phenylalanine	38 [22]	12	07	18
MSUD	Valine, Isoleucine, Leucine	17 (10.1)	01	04	12
NKHG	Glycine	15 (8.9)	04	04	07
HCY	Methionine	13 (7.7)	01	03	09
GLY	Glycine	12 (7.14)	–	06	06
CIT-I	Citrulline	08 (4.76)	02	05	01
CPS	Alanine	03 (1.8)	01	02	–
LIVER	Methionine, Tyrosine	02 (1.2)	–	2	–
GLN	Glutamine	01 (0.6)	–	01	–
OCT (het)	Glutamine, Alanine	01 (0.6)	–	–	01
TYR	Tyrosine	01 (0.6)	–	–	01
BCAA LOW	–	03 (1.8)	–	–	–
LPI	–	02 (1.2)	–	–	–
MTHFR	–	01 (0.6)	–	–	–
PRO	–	02 (1.2)	–	–	–
MET LOW	–	02 (1.2)	–	–	–
PA	–	02 (1.2)	–	–	–

***Legend:****Possibly, likely and very likely represent the likelihood of the disorder. CIT-I, Citrullinemia-I; CPS, carbamoyl phosphate synthetase I deficiency; GLN, Possible hyperammonemia; GLY, Elevated glycine; HCY, homocystinuria; LIVER, Undetermined liver disease*; *MSUD, Maple syrup urine disease; NKHG, Non-Ketotic hyperglycinemia; NON-FAST, Nonfasting specimen; OTC (het), Heterozygote for ornithine transcarbamylase deficiency; PKU, Phenylketonuria; TYR, elevated tyrosine; BCAA LOW, low branched-chain amino acids; LPI, Lysinuric Protein Intolerance; MTHFR, Methylenetetrahydrofolate reductase; PO, Elevated proline; MET LOW, Possible remethylation disorder; PA, Propionic Acidemia.*

A concordance of 98.8% was noted between the reporting done by the BGL and then after applying CLIR tools. Among 168 cases, one case was labeled as marked elevation noted in branched-chain amino acids (BCAA) and another case was labeled as marked glycine (GLY) at BGL of our institute while CLIR labeled those cases as MSUD and NKHG respectively, details of both cases are described in Table 3. Of note, not all tools in CLIR are specific for a single underlying metabolic disorder. Some, such as those for non-fasting samples or liver disease are designed to identify common deviations from the reference population, but they are not diagnostic. These tools for targeted conditions and common abnormal profiles were able to identify a pattern in each profile that was not normal according to local reference ranges.

**Table 3:** Description of discordant results (n = 2) when all conditions tool from CLIR was applied.

Case number	Age at the collection in years	Gender	Diagnosis by authors	Diagnosis as per CLIR	Resolution by All condition tool	Percentile Rank of Case Scores	The score for the disease	Guidelines for scoring
103	0.0575	Male	Markedly elevated glycine	Non-Ketotic Hyperglycinemia (NKHG)	Informative	58.46%	171	Condition is likely NKHG (score points >130 and < 260)
				Elevated glycine (GLY)	Informative	74.78%	181	Condition is likely GLY (score points >50 and <195)
134	02	Female	Elevated branched-chain amino acids	Maple Syrup Urine Disease (MSUD)	Informative	8.58%	40	Condition is possibly MSUD (score points >5 and < 90)
				Branched-chain amino acid (BCAA)	Non- Informative	0	0	Not informative for BCAA

**Fig. 3:**
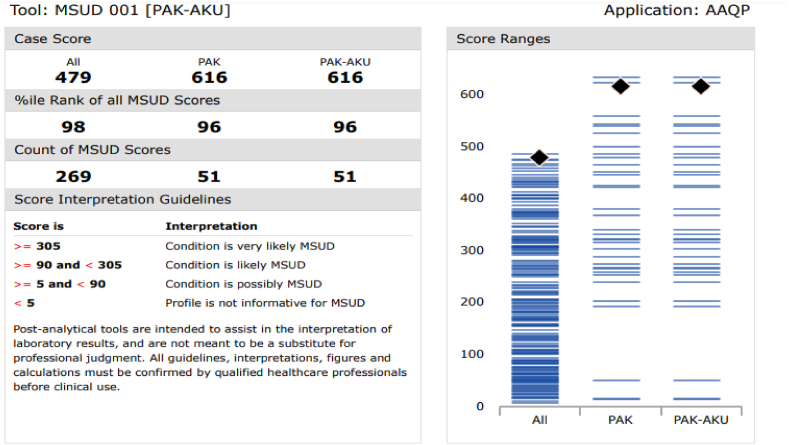
CLIR Single condition tool score interpretation guidelines & Score Ranges Chart for MSUD (shown as an example). *Legend:****Right****, Score Ranges chart is a visual representation of all active Cases with the Condition the Tool is based on. Each Case Score from the Case database is shown as a blue line. Case Scores are displayed for the cumulative project (All), the Aggregator group (PAK), and the designated Location (PAK-AKU). The Case Score of the case of interest is represented on the chart using black diamonds***. *Left****, The Case Score table provides the Case Score, %ile Rank of the case under review compared to all Cases with the specified Condition, and the count of scores available for the specified Condition. The Score Interpretation Guidelines section of the Score Report is a textual description of Tool interpretation guidelines.* . (For interpretation of the references to color in this figure legend, the reader is referred to the Web version of this article.)

Each metabolic disorder was then studied in detail using the single condition tool and longitudinal plots. Taking the example of MSUD, on the application of a single condition tool on CLIR, out of the total 17 MSUD cases shown in Fig. 3, based on CLIR scoring guidelines 12 were identified as very likely (scores ≥305), four were identified as likely MSUD (scores ≥90 and < 305) and one possibly MSUD (scores ≥5 and < 90) respectively. Fig. 3 shows the total number of MSUD cases diagnosed by our clinical laboratory and uploaded to CLIR since 2018 including a total of 17 cases from the current study.

**Fig. 4:**
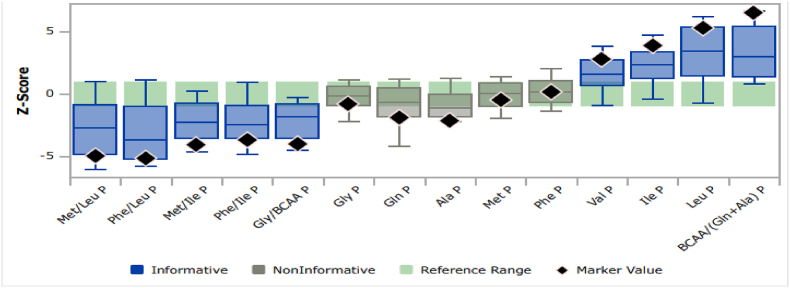
CLIR Single condition tool for MSUD showing reference percentile and disease range overlap plot. ***Legend:****The abbreviated names of each Marker are shown on the X-axis. The Y-axis is a logarithmic expression of the reference and disease-specific ranges converted to a Z-Score. A green box depicts the 1–99%ile of the reference population for each Marker. A box plot depicts the* 1st*,* 10th*, median,* 90th*, &* 99th *percentiles for the disease range of each Marker. Informative marker, meaning the median of the disease range is above the 99%ile or below the 1%ile of the reference population, in the plot is colored blue, Non-Informative Marker is plotted grey. The Marker Values for the case under review are shown on the plot with black diamonds*. . (For interpretation of the references to color in this figure legend, the reader is referred to the Web version of this article.)

In Fig. 4 the Plot by Condition portion of the Single Condition Tool provides a visual display of how closely this case of MSUD matches the pattern of the Marker disease ranges unique to MSUD. In this plot, logarithmic expression of the reference and disease-specific ranges converted to a Z-Score for all analyte values are shown on the y axis and the disease ranges are expressed not as absolute results but are first converted to a multiple of the median value from the cumulative reference population of the entire project. Each column represents a single marker, in this plot, only PAA are shown as per the selection made on CLIR relevant to our study objective.

## 4 Discussion

In this study, we calculated plasma levels for 22 amino acids from the 1913 reference population, boys (56.2%) and girls (43.8%), with a median age of one year. Except for cystine, glutamine, and valine, all other PAA were in close comparison to cumulative reference range medians available in the CLIR database. The reason for low levels might be because of low protein intake secondary to illness. Results from our study also showed that using CLIR tools different cases of aminoacidopathies were correctly identified by the BGL team at our institute with 98.8% concordance. Two experts reviewed this PAA profile along with biochemical and clinical provided data. For confirmation of disorder, further investigation was not made for these two discordant cases because of financial constraints. As the knowledge and expertise to understand BGL reports is minimal in our country therefore, providing the accurate diagnosis of these rare disorders MSUD and NKH respectively would have helped the physicians managing the patients. Low rare disease awareness among physicians is believed to be one of the reasons for late and misdiagnoses of rare disease patients in this part of the world [17]. The results of PAA supplemented by the interpretation provided by the CLIR tools assisted in providing the strongest basis for the decision on reporting. Our results do not represent the full discriminatory power of the analytical tools in CLIR, predominantly the dual scatter plot, to further distinguish between true positives and carriers’ confirmatory tests that were not performed in the current study population. Our data clearly show the disorders for which the CLIR tools significantly support decision-making based on the PAA concentration in the high-risk patient population. The median of the majority PAA profile was close to the 50th percentile of reference data from CLIR except for aspartate with median 3 times greater than the reference, which might be secondary to late sample analysis or in vivo hemolysis.

Most IMDs are complex disorders that cannot be modeled with simple parametric distributions. CLIR tools are meant to recognize the different patterns in PAA to improve sensitivity and specificity. This post-analytical software does not rely on the traditional definition of “abnormal” as merely a deviation from a normal reference range [18,19]. Rather, it places patients within condition-specific disease ranges and evaluates how consistent a result is with the analyte disease range established separately for each condition [10,16]. Multiple studies have utilized CLIR-post-analytical tools or machine learning algorithms on newborn screening data using dried blood spots, and then following the patients with clinical outcomes and targeted diagnostics. The positive predictive value for different IMDs was increased from 26% to 54% using CLIR along with second-tier biochemical testing on newborn screening data from Norway [20]. The employment of various post-analytical CLIR tools improved the timely identification of preventable false positive's cases and the follow-up burden for patients and their families [16,[21], [22], [23], [24]]. This is one of the major advantages of using CLIR in reporting PAA results. It may provide confidence in reporting and interpretation of the PAA results as shown in the current study. Many aminoacidopathies are complex and difficult to diagnose with many of the disorders being exceptionally rare [25,26]. Issues in Pakistan are complicated with few experienced scientists and pathologists. Diagnosis of aminoacidopathies in Pakistan has been difficult due to resource constraints and limited clinical and technical proficiency. Before 2012, all diagnostic tests were outsourced, as there was no local diagnostic facility [27,28]. As currently there is no newborn screening program in the country most of the patients are either diagnosed incidentally or late when they become symptomatic. The diagnostic challenges multiply in a country where the national newborn screening program is non-existent, and the data on positive cases is limited. The patient data uploaded in the current study were all high-risk and mostly symptomatic. In our country even biochemical genetics laboratory facilities are scarce and no national registry or biorepository for aminoacidopathies. Validating these positive PAA results with CLIR and giving interpretations with clarity is useful and builds confidence in our program. In the future, the plan is to apply CLIR in our hospital-based expanded newborn screening program for congenital hypothyroidism, congenital adrenal hyperplasia, and IMDs.

## 5 Limitation

The data available in CLIR is from PAA analyzed using tandem mass spectrometry (MS/MS) while PAA analyzed on BIOCHROM (HPLC). This would add to bias due to differences in analytical methodologies. However, our BGL has been successfully participating in CAP and ERNDIM external quality assurance schemes. More limitations of the current study were a cross-sectional study design with no follow-up of positive patients. Second-tier testing was not performed for a few IMDs such as alloisoleucine for MSUD, tetrahydrobiopterin, or enzyme analysis for phenylketonuria, etc. The dietary and nutritional intake of subjects was not evaluated hence the association of PAA with diet could not be evaluated. It would be interesting to examine whether PAA concentrations vary with the nutritional status of individuals and growth outcomes.

## 6 Conclusion

More than ninety-eight percent of aminoacidopathies were correctly identified using CLIR tools. With the use of this software, it has become readily possible to rapidly screen the whole spectrum of calculated ratios across all markers. This approach has the potential to produce a more comprehensive and accurate explanation of difficult laboratory profiles, driven by multisite evidence and by peer comparison. The high concordance in this study is a testimony of accurate laboratory preanalytical, analytical, and post-analytical processes. In the future, the application of CLIR tools can be utilized in a newborn screening program for screening and diagnosis of congenital hypothyroidism, congenital adrenal hyperplasia, and even IMDs in our local setup.

## Ethical approval

The study was carried out after seeking approval from Aga Khan University ethical review committee (Reference number 2019-1709-4536.

## Source of funding

None declared.

## Author contribution

The conception, design of the study and interpretation of data were performed by Lena Jafri**;** Acquisition of data, analysis & interpretation of data and drafting the article were performed by Zaib Un Nisa Khan and Lena Jafri**;** Revision of manuscript critically for important intellectual content were performed by Hafsa Majid, Aysha Habib Khan, Sibtain Ahmed, Patricia L Hall, Matthew J Schultz**;** Final approval of the version to be submitted was done by all authors.

## Trail registry number

Name of the registry: ClinicalTrials.gov.

Unique Identifying number or registration ID: NCT05437445.

Hyperlink to your specific registration (must be publicly accessible and will be checked): https://register.clinicaltrials.gov/prs/app/action/ViewOrUnrelease?uid=U0005Z5D&ts=3&sid=S000C93M&cx=-ck0wpy.

## Guarantor

Dr Lena Jafri, llena.jafri@aku.edu. Corresponding author.

## Availability of data and materials

The datasets generated and/or analyzed during the current study are not publicly available due to privacy restrictions but are available from the corresponding author upon reasonable request.

## Provenance and peer review

Not commissioned, externally peer reviewed.

## Declaration of competing interest

Authors state no conflict of interest.
